# Vaccinating and non-vaccinating parents' attitudes toward influenza vaccination in children under 5 years old in Spain

**DOI:** 10.3389/fpubh.2025.1644600

**Published:** 2025-10-01

**Authors:** Matilde Zornoza-Moreno, Susana Sánchez-Manresa, María Cruz Gómez-Moreno, María Pilar Ros Abellán, Jaime J. Pérez-Martín

**Affiliations:** Prevention and Health Protection Service, Regional Ministry of Health, Murcia, Spain

**Keywords:** influenza, vaccine, intranasal, intramuscular, parents, satisfaction

## Abstract

**Background:**

Vaccines against seasonal influenza represent the best option to reduce the cases and severity of the disease in children under 5 years. In Spain, the Region of Murcia was a pioneer in implementing universal vaccination at schools. The study aimed to evaluate the acceptance and attitudes of parents of children of 6–59 months old toward influenza vaccination and factors associated with parents' decision to vaccinate their children during the 2023–2024 campaign.

**Methods:**

This prospective, descriptive study used a questionnaire to collect data from parents of vaccinated (VC) and non-vaccinated children (nVC). VC received either the live-attenuated intranasal vaccine (LAIV) or the intramuscular vaccine (IIV) depending on their age.

**Results:**

Parent's self-vaccination/intention in this campaign (OR: 8.51) and in the previous one (OR: 4.49), and children's compliance with vaccination schedule (OR: 7.83) were the main factors associated with the probability of children being vaccinated against influenza. The main reason for vaccinating was to protect the child (IIV: 92.5% vs. LAIV: 91.0%), while waiting for further experience with the vaccine (21.0%) and lack of recommendation from the healthcare professional (20.4%) were the major reasons for not vaccinating. Most vaccinating parents (IIV: 90.4% vs. LAIV: 93.9%, *p* < 0.001) were satisfied with the vaccine. Among VC at school, 95.8% of parents were satisfied and 97.4% would recommend for family and friends.

**Conclusions:**

Parents of VC, especially those vaccinated with LAIV at schools, were highly satisfied with the vaccine. Parent's influenza vaccination status and children's compliance with vaccination schedule were determinant for parent's decision to vaccinate children under 5 years old. More information about influenza vaccination could help increase vaccination rates in these children.

## 1 Introduction

Seasonal influenza is a common cause of respiratory infection during cooler and lower humidity winter months (1). It is estimated that the annual seasonal influenza epidemic causes one billion cases worldwide, resulting in 3–5 million cases of severe disease and up to 650,000 deaths (2). Children under the age of 5 years old are among those with greater risk of severe disease, hospitalization, and death, accounting for approximately 35,000 deaths annually (2, 3). In Spain, the highest influenza incidence during the 2022–2023 seasons was observed in children of 0–4 and 5–14 years old. Similarly, children of 0–4 years old presented the second highest hospitalization rate only after people aged 80 years or older (4).

As influenza spreads easily among people, vaccination represents the best strategy to prevent the disease (2). In fact, the World Health Organization (WHO) and the European Centre for Disease Prevention and Control (ECDC) included in 2012 children aged 6 to 59 months among those who should be vaccinated (5, 6). In Spain, this recommendation was later introduced in 2022 by the National Immunization Technical Advisory Group (NITAG) (7). Additionally, the Advisory Committee on Vaccines and Immunizations of the Spanish Association of Pediatrics (CAV-AEP) included the preferential use of the live attenuated vaccines (LAIV) in children from the age of 2 years and the intramuscular inactivated vaccines (IIV) in those of 6 to 23 months (8, 9).

Despite the importance of vaccination and the recommendations issued by different authorities, pediatric vaccination rates against influenza are still low (10–12). In Spain, during the 2022–2023 seasons, three autonomous communities (Andalusia, Galicia, and the Region of Murcia) implemented universal vaccination against influenza in children of 6 to 59 months, with vaccination rates ranging from 45.1% to 54.2%. For the next season (2023–2024), the first one at national level, the mean vaccination coverage in children of 1 to 4 years of age was 37.2%, although high disparity between autonomous communities has been observed (13). In the Region of Murcia, during the 2022–2023 campaign, a pilot experience on school vaccination was conducted with children aged 3 and 4 years old (14), being extended to all schools for the 2023–2024 vaccination campaign (15).

Vaccine hesitancy and parents' knowledge and attitudes toward vaccination could significantly influence whether or not their children receive the vaccine against influenza. In this sense, a recent systematic literature review and meta-analysis analyzed the prevalence for parental acceptance, hesitancy and uptake of seasonal influenza vaccination in children of 6 to 59 months and reported rates of 64%, 34%, and 41%, respectively. In addition, the authors identified several factors associated with acceptance or hesitancy which included perceived benefits and barriers, among others (16).

In Spain, the FLUTETRA study was designed and implemented to collect data on the profile of parents of vaccinated (VC) and non-vaccinated children (nVC) during the first vaccination campaign in the Region of Murcia, the 2022–2023, showing that parents' self-vaccination or intention to be vaccinated was associated with children's vaccination. Regarding non-vaccinating parents, the main reasons for not vaccinating their children were the lack of recommendation from healthcare professionals and the lack of information on the vaccine and the vaccination campaign (17).

In this context, and with the intention of continuing the analysis started during the first campaign, the aim of this study was to evaluate the acceptance and attitudes of parents of children of 6 to 59 months of age toward influenza vaccination, for those of 3 and 4 years old vaccinated at schools, and factors associated with parents' decision to vaccinate during the second vaccination campaign, the 2023–2024. In addition, we aimed to describe the sociodemographic characteristics of parents of VC and nVC, as well as the attitude and reason for refusing vaccination in parents of nVC during a second influenza vaccination campaign.

## 2 Methods

### 2.1 Study design

This is a prospective descriptive study conducted during the 2023–2024 influenza vaccination season to evaluate the attitude of parents or legal representatives of children of 6 to 59 months of age toward influenza vaccination in the Region of Murcia (Spain) using an online questionnaire specifically designed for this purpose.

Parents of VC with an influenza dose registered in VACUSAN (data management program of the Region of Murcia vaccination registry) from September 25, 2023 to February 29, 2024 were invited to participate in the study, receiving the survey 7 days after vaccine administration. Parents of nVC from 6 to 59 months of age were enrolled after the end of the vaccination campaign (April 19, 2024). Upon acceptance, parents could access the study questionnaire through a link sent by text message sent to the main contact telephone number available in the vaccinated child's file.

The study was approved by the Ethics Committee of the Hospital Clínico Universitario Virgen de la Arrixaca and conducted following the Good Clinical Practice Guidelines and the Declaration of Helsinki. In accordance with local regulations, the Ethics Committee approved an exemption from written informed consent.

### 2.2 Study questionnaire

The study questionnaires directed to parents of vaccinated and non-vaccinated children contained 39 and 32 questions, respectively, collecting information on sociodemographic and clinical characteristics of children and parents and parents' attitudes toward influenza vaccination (Supplementary material).

The questions related to participants' demographic characteristics were dichotomic (yes or no) or had predefined options. The level of agreement with questions related to the satisfaction with the vaccines were assessed using a 5-point Likert scale where a score of 1 indicated the worst/lowest (strongly disagree) available option and a score of 5 was associated with the best/highest (strongly agree) available option. Questions related to reasons for not vaccination had predefined options.

### 2.3 Treatment

Vaccination was carried out following the local standard of care (18). Briefly, children of 6 to 23 months received Influvac^®^ Tetra IIV (Mylan IRE Healthcare Limited). Children of 24 to 59 months received Fluenz^®^ Tetra LAIV (AstraZeneca UK Limited), except those with specific contraindications to LAIV, who received IIV Flucelvax Tetra^®^ (Seqirus Netherlands B.V.). With parental consent, 3 and 4-years old children were vaccinated at school.

### 2.4 Statistical analysis

Demographic and clinical characteristics were described using central (mean) and dispersion [standard deviation (SD)] measures for quantitative variables and absolute (N) and relative (%) frequencies, along with 95% confidence interval (CI), for qualitative variables.

The comparison between VC and nVC and between vaccine types was assessed using the non-parametric Mann-Whitney U-test for quantitative variables and the Chi-square or Fisher's exact test for qualitative variables.

A multivariable logistic regression model was used to assess the potential association of factors with parents' decision to vaccinate. Variables included in the model (prematurity, number of siblings, parent's vaccination against influenza in both current and past campaigns, presence of acute infection disease during the previous year, children's up-to-date vaccination schedule, administration of non-funded vaccines, children's vaccination against influenza in the last campaign, parent's chronic disease, cohabitants with chronic disease and/or 60-years of age and older, and parent's opinion on childhood diseases causing most hospitalizations) were those with a *p-value* < 0.2 in the prior univariate analysis. Odds ratio (OR) and 95% CI were calculated.

All statistical analyses were performed using the SAS v9.4 software through the SAS Enterprise Guide v8.3 interface. All *p*-*values* < 0.05 were considered statistically significant.

## 3 Results

### 3.1 Characteristics of the study population

The invitation to participate in the study was sent to 64,243 parents, 25,411 parents of VC (7,658 and 17,753 children received IIV and LAIV, respectively), and to 38,832 parents of nVC.

A total of 5,224 (8.1%) parents or legal representatives of children aged 6–59 months responded the survey and were included in the study. Of these, 4,251 (81.4%) children were vaccinated against influenza during the 2023–2024 vaccination campaign (response rate of 20.6%), while 973 (18.6%) children were not vaccinated (2.5% of response rate).

Overall, children were nearly equally distributed by sex and >95% of them were Spanish. Vaccinated children showed a higher proportion of prematurity (8.2% vs. 6.0%, *p* = 0.02) and history of acute infectious disease requiring medical attention during 2023 (43.4% vs. 39.9%, *p* = 0.048) compared with nVC. In addition, VC presented higher compliance with vaccination schedule according to their age compared with nVC (99.8% vs. 97.0%, *p* < 0.001) (Table 1).

**Table 1:** Characteristics of children and parents included in the study stratified by children's vaccination status.

**Characteristic**	**Vaccinated children (*N* = 4,251)**	**Non-vaccinated children (*N* = 973)**	***p*-*value***
**Children's characteristics**			
Age, *n* (%)			< 0.001
6–11 months	476 (11.2)	128 (13.2)	
12–23 months	783 (18.4)	166 (17.1)	
2 years	714 (16.8)	252 (25.9)	
3 years	1,144 (26.9)	162 (16.6)	
4 years	1,134 (26.7)	265 (27.2)	
Sex (female), *n* (%)	2,092 (49.2)	455 (46.8)	0.177
Premature birth (< 37 weeks), *n* (%)	347 (8.2)	58 (6.0)	0.02
Country of origin, *n* (%)			< 0.001
Spain	4,196 (98.7)	932 (95.8)	
Other	55 (1.3)	41 (4.2)	
Siblings, *n* (%)			0.393
0	1,627 (38.3)	378 (38.9)	
1	1,980 (46.6)	430 (44.2)	
2	483 (11.4)	127 (13.1)	
>2	161 (3.8)	38 (3.9)	
History of acute infectious disease requiring medical attention in 2023, *n* (%)	1,843 (43.4)	388 (39.9)	0.048
Complete vaccination schedule according to age, *n* (%)	4,241 (99.8)	944 (97.0)	< 0.001
Non-funded vaccines, *n* (%)	3,644 (85.7)	717 (73.7)	< 0.001
Influenza vaccination in last campaign (2022–2023), *n* (%)	2,466 (58.0)	188 (19.3)	< 0.001
Chronic disease, *n* (%)	203 (4.8)	45 (4.6)	0.933
**Parents characteristics**			
Age, *n* (%)			< 0.001
< 20 years	8 (0.2)	5 (0.5)	
20–29 years	293 (6.9)	118 (12.1)	
30–39 years	2,485 (58.5)	517 (53.1)	
40–49 years	1,422 (33.5)	320 (32.9)	
>50 years	43 (1.0)	13 (1.3)	
Sex (female), *n* (%)	3,861 (90.8)	827 (85.0)	< 0.001
Country of origin, *n* (%)			< 0.001
Spain	3,716 (87.4)	800 (82.2)	
Other	535 (12.6)	173 (17.8)	
Education			0.017
None	25 (0.6)	7 (0.7)	
Primary education	290 (6.8)	53 (5.5)	
Secondary education	1,518 (35.7)	397 (40.8)	
Higher education	2,418 (56.9)	516 (53.0)	
Chronic disease, *n* (%)	745 (17.5)	168 (17.3)	0.888
Parent's influenza vaccination in previous campaign (2022–2023), *n* (%)	2,023 (47.6)	221 (22.7)	< 0.001
Parents' influenza vaccination (or intention) in the current campaign (2023–2024), *n* (%)	2,092 (49.2)	82 (8.4)	< 0.001
Cohabitants with chronic disease and/or older than 60 years, *n* (%)	547 (12.9)	102 (10.5)	0.046
**Parent's opinion about childhood diseases causing most**			
**hospitalizations**			
Rotavirus gastroenteritis	397 (9.3)	135 (13.9)	< 0.001
Influenza	1,464 (34.4)	190 (19.5)	< 0.001
Meningitis	305 (7.2)	93 (9.6)	0.0132
Pneumonia	2,080 (48.9)	550 (56.5)	< 0.001
Measles	5 (0.1)	5 (0.5)	0.024

Regarding the characteristics of the parents/legal representatives who responded the survey, most of them were females (VC: 90.8% vs. nVC: 85.0%, *p* < 0.001) from Spain (VC: 87.4% vs. nVC: 82.2%, *p* < 0.001), with a higher percentage in VC group. A higher proportion of parents of VC received the vaccine against influenza during last season (47.6% vs. 22.7%, *p* < 0.001) and were currently vaccinated or had the intention to be vaccinated during the present campaign (49.2% vs. 8.4%, *p* < 0.001) compared with parents of nVC. Furthermore, a higher proportion considered that influenza is a childhood disease causing the higher number of hospitalizations (34.4% vs. 19.5%, *p* < 0.001). In contrast, more parents of nVC considered that pneumonia causes the higher number of hospitalizations (56.5% vs. 48.9%, *p* < 0.001) (Table 1).

Among all VC, 1,259 (29.6%) and 2,992 (70.4%) children received the IIV and LAIV, respectively.

The comparison between vaccination groups showed statistically significant differences regarding children's age, country of origin, non-funded vaccines received, influenza vaccination in last campaign, and chronic diseases (Table 2). A higher proportion of parents of children receiving the IIV vs. LAIV were vaccinated against influenza during previous campaign (58.3% vs. 43.1%, *p* < 0.001), and in the current or had the intention to be vaccinated (51.6% vs. 48.2%, *p* = 0.044) (Table 2).

**Table 2:** Characteristics of vaccinated children and their parents according to the vaccine received.

**Characteristic**	**IIV (*N* = 1,259)**	**LAIV (*N* = 2,992)**	***p*-value**
**Children characteristics**			
Age, *n* (%)			< 0.001
6–11 months	476 (37.8)	–	
12–23 months	783 (62.2)	–	
2 years	–	714 (23.9)	
3 years	–	1,144 (38.2)	
4 years	–	1,134 (37.9)	
Sex (female), *n* (%)	606 (48.1)	1,486 (49.7)	0.365
Premature birth (< 37 weeks), *n* (%)	111 (8.8)	236 (7.9)	0.326
Country of origin, *n* (%)			< 0.001
Spain	1,255 (99.7)	2,941 (98.3)	
Other	4 (0.3)	51 (1.7)	
History of acute infectious disease requiring medical attention in 2023, *n* (%)	541 (43.0)	1,302 (43.5)	0.760
Complete vaccination schedule according to age, *n* (%)	1,254 (99.6)	2,987 (99.8)	0.173
Non-funded vaccines, *n* (%)	1,114 (88.5)	2,530 (84.6)	< 0.001
Influenza vaccination in last campaign (2022–2023), *n* (%)	404 (32.1)	2,062 (68.9)	< 0.001
Chronic disease, *n* (%)	47 (3.7)	156 (5.2)	0.040
**Parents characteristics**			
Age, *n* (%)			< 0.001
< 20 years	4 (0.3)	4 (0.1)	
20–29 years	122 (9.7)	171 (5.7)	
30–39 years	827 (65.7)	1,658 (55.4)	
40–49 years	300 (23.8)	1,122 (37.5)	
>50 years	6 (0.5)	37 (1.2)	
Sex (female), *n* (%)	1,124 (89.3)	2,737 (91.5)	0.027
Country of origin, *n* (%)			0.06
Spain	1,094 (86.9)	2,622 (87.6)	
Other	165 (13.1)	370 (12.4)	
Education			< 0.001
None	8 (0.6)	17 (0.6)	
Primary education	58 (4.6)	232 (7.8)	
Secondary education	420 (33.4)	1,098 (36.7)	
Higher education	773 (61.4)	1,645 (55.0)	
Chronic disease, *n* (%)	213 (16.9)	532 (17.8)	0.508
Influenza vaccination in previous campaign (2022–2023), *n* (%)	734 (58.3)	1,289 (43.1)	< 0.001
Parents' influenza vaccination (or intention) in the current campaign (2023–2024), *n* (%)	650 (51.6)	1,442 (48.2)	0.044
Cohabitants with chronic disease and/or older than 60 years, *n* (%)	159 (12.6)	388 (13.0)	0.802

IIV, intramuscular injected vaccine; LAIV, live-attenuated intranasal vaccine.

### 3.2 Factors associated with vaccination against influenza

The multivariable logistic regression identified parent's influenza vaccination (or intention) in this campaign (OR: 8.51), and in the previous one (OR: 4.49), as well as children's complete vaccination schedule according to their age (OR: 7.83) as the main factors associated with the probability of children being vaccinated against influenza (Table 3).

**Table 3:** Factors associated with the probability of vaccination against influenza in children aged 6 to 59 months identified in the multivariable analysis.

**Variable**	**OR (95% CI)**	***p*-*value***
Parent's influenza vaccination (or intention) in this campaign	8.51 (6.67–10.86)	< 0.001
Complete vaccination schedule according to children's age	7.83 (3.36–18.28)	< 0.001
Parent's influenza vaccination in the previous year	4.49 (3.75–5.38)	< 0.001
Administration of non-funded vaccines	1.69 (1.38–2.07)	< 0.001
Parent's chronic disease	0.79 (0.64–0.97)	0.028
**Parent's opinion about childhood diseases causing the higher**		
**number of hospitalizations vs. influenza**		
Rotavirus gastroenteritis	0.22 (0.16–0.28)	< 0.001
Meningitis	0.34 (0.25–0.48)	< 0.001
Pneumonia	0.39 (0.31–0.48)	< 0.001
Measles	0.11 (0.02–0.57)	0.009

CI, confidence interval; OR, odds ratio.

### 3.3 Reasons for vaccination and satisfaction with vaccines

**Figure 1:**
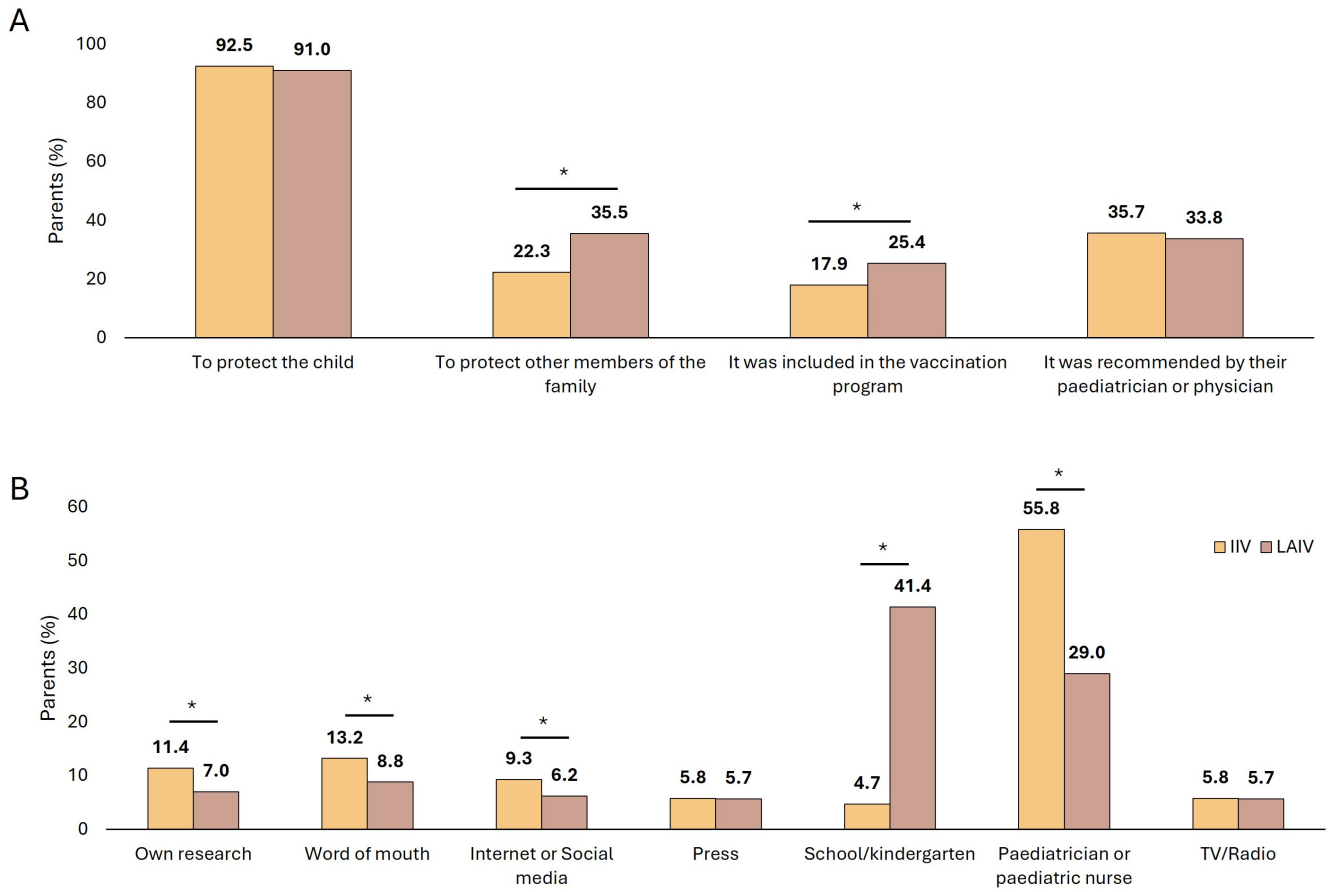
Proportion of parents of vaccinated children reporting reasons for vaccination **(A)** and source of information **(B)** depending on the vaccine received. IIV, injectable intramuscular vaccine; LAIV, live-attenuated intranasal vaccine. *p < 0.001.

When asked about the main reason for vaccinating their children, 91.4% of parents, regardless of the vaccine administered, responded that it was “to protect the child.” Other reasons included “it was recommended by their pediatrician of physician” (34.3%), “to protect other members of the family” (31.6%; LAIV: 35.5% vs. IIV: 22.3%, *p* < 0.001) and “due to inclusion in the vaccination program” (23.2%; LAIV: 25.4% vs. IIV: 17.9%, *p* < 0.001), with the two latter being selected by a higher proportion of parents of children vaccinated with LAIV compared with those vaccinated with IIV (Figure 1A).

On the other hand, the most common source of information for parents of children vaccinated with IIV was the pediatrician or pediatric nurse (IIV: 55.8% vs. LAIV: 29.0%, *p* < 0.001) while for parents of children vaccinated with LAIV was the school or kindergarten (LAIV: 41.4% vs. IIV: 4.7%, *p* < 0.001) (Figure 1B).

**Figure 2:**
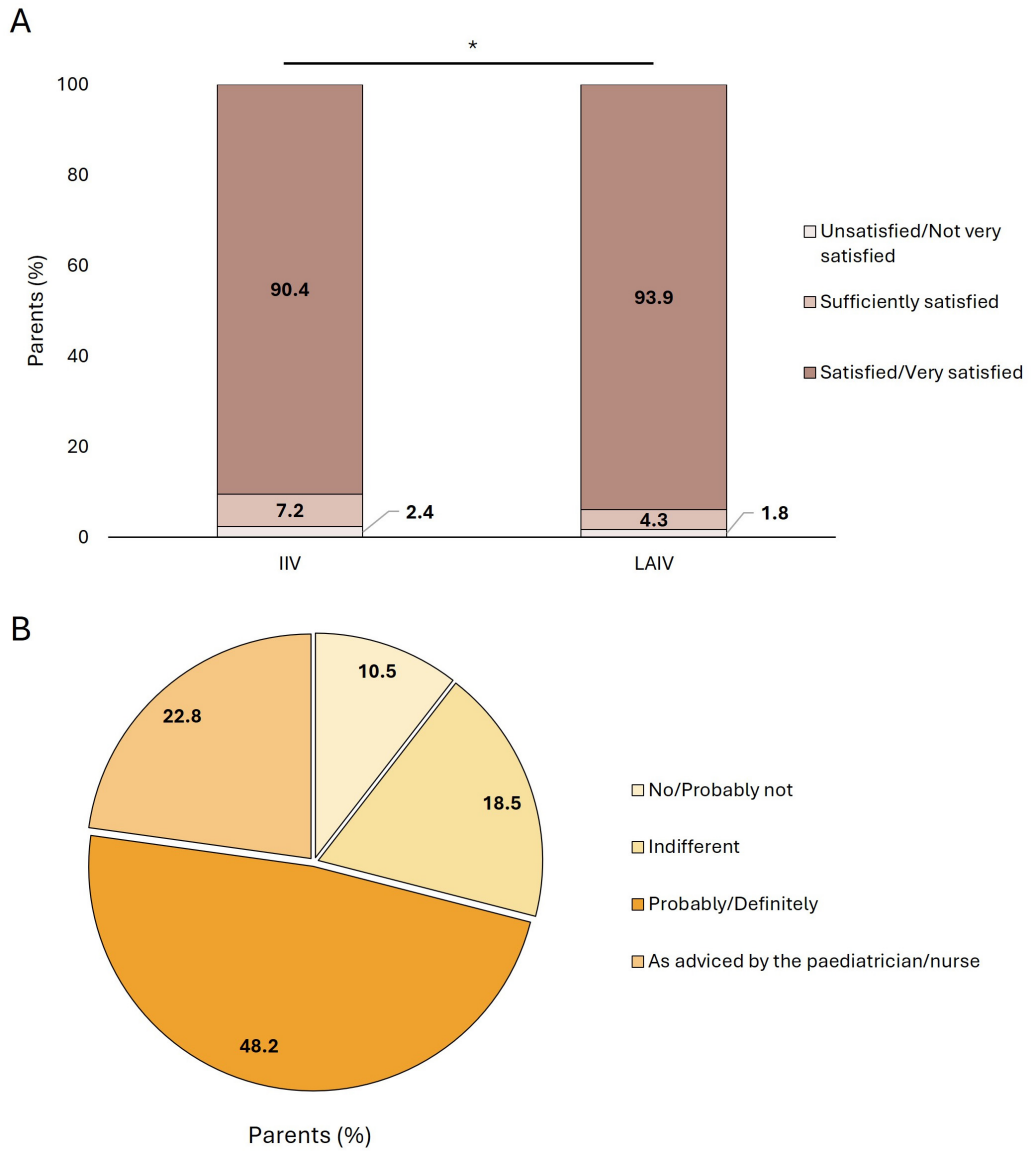
Satisfaction of parents of vaccinated children with the vaccine administrated stratified by vaccine type **(A)** and perspectives of parents of children vaccinated with the IIV for future vaccination campaigns **(B)**. IIV, injectable intramuscular vaccine; LAIV, live-attenuated intranasal vaccine. *p < 0.001.

Most parents of VC were satisfied or very satisfied with both vaccines; despite the fact that statistically significant differences were observed between vaccination groups (IIV: 90.4% vs. LAIV: 93.9%, *p* < 0.001) (Figure 2A).

Parents of children vaccinated with IIV were asked about using LAIV for the next campaign and almost half of them (48.2%) responded that they would probably or definitely vaccinate their children with the LAIV (Figure 2B).

Overall, most parents of VC had the intention to vaccinate their children during the next influenza season (86.5%) and would recommend vaccinating against influenza for the children of family and friends (97.4%).

Parents of children vaccinated with LAIV indicated that 40.4% and 59.6% were vaccinated in the Health Centre and at school, respectively. Of those vaccinated at school, 95.8% of parents were satisfied or very satisfied with LAIV vaccination. In addition, 88.4% mentioned that if the vaccine had been IIV, they would have still vaccinated their children.

### 3.4 Reasons for non-vaccination

Overall, parents of nVC declared that waiting for further experience with the flu vaccine (21.0%) and the lack of recommendation from the healthcare professional of reference (20.4%) were the main reasons for not vaccinating their children (Table 4).

**Table 4:** Reasons for non-vaccination stratified by children's age.

**Reason**	**Total (*N* = 973)**	**6–23 months (*N* = 294)**	**24–59 months (*N* = 679)**	***p*-*value***
I would rather wait until further experience with the flu vaccine	204 (21.0)	59 (20.1)	145 (21.4)	0.669
The healthcare professional of reference did not recommend it	198 (20.4)	80 (27.2)	118 (17.4)	< 0.001
Lack of information about the vaccination campaign	152 (15.6)	64 (21.8)	88 (13.0)	< 0.001
Lack of information about the vaccine	145 (14.9)	53 (18.0)	92 (13.6)	0.078
My son/daughter has contracted flu this year	134 (13.8)	42 (14.3)	92 (13.6)	0.762
The vaccine did not demonstrate effectiveness	127 (13.1)	25 (8.5)	102 (15.0)	0.005
I consider flu an infection of minor importance in children < 5 years	122 (12.5)	30 (10.2)	92 (13.6)	0.171
Inaccessibility (i.e., impossibility to get an appointment)	92 (9.5)	15 (5.1)	77 (11.3)	0.002
I consider the flu vaccine unsafe	89 (9.2)	20 (6.8)	69 (10.2)	0.115
Influence of friends'/family's opinion	57 (5.9)	21 (7.1)	36 (5.3)	0.298
I do not believe in vaccines	39 (4.0)	12 (4.1)	27 (4.0)	1.0

A higher proportion of parents of children aged 6–23 months declared that there was a lack of information about the vaccination campaign (21.8% vs. 13.0%, *p* < 0.001) and a lack of recommendation from the healthcare professional of reference (27.2% vs. 17.4%, *p* < 0.001) compared with parents of children aged 24–59 months. On the other hand, more parents of children of 24–59 months declared that the vaccine did not demonstrate effectiveness (15.0% vs. 8.5%, *p* = 0.005) compared with parents of younger children (Table 4).

## 4 Discussion

The present study expands the available knowledge from the previous campaign (year 2022–2023) about the opinion of parents of VC and nVC to help understand the paradigm of vaccination against influenza in the Region of Murcia. Additionally, to the best of our knowledge, our study is one of the few published studies showing parental support to school vaccination. The obtained results could help provide more accurate information for parents who decided not to vaccinate, ultimately helping to improve influenza vaccination rates in future campaigns.

The influenza vaccination rate in children under 4 years during the 2023–2024 campaign at national level was 37.2%, while in the Region of Murcia was 51.5%. Despite no specific targets for vaccine uptake were set for this campaign, as this was the first one at national level, vaccination rates in the Region of Murcia increased from 45.1% during the 2022–2023 campaign (13).

The results of our study showed that, overall, VC presented a higher proportion of prematurity, history of acute infectious disease requiring medical attention during 2023, complete vaccination schedule, and administration of non-funded vaccines in comparison with nVC. In contrast, despite previous studies have shown that higher numbers of siblings were associated with lower vaccination rates (19), in the present study no differences between VC and nVC were observed regarding the number of siblings. In addition, we found significant differences in children's vaccination status depending on the children's and parent's country of origin, with a higher proportion of VC and their parents born in Spain. This is in line with what we observed during last vaccination campaign against influenza (17) and in the first vaccination campaign against respiratory syncytial virus with nirsevimab (20), which might suggest that language barriers in immigrants would results in lower vaccination rate. Moreover, a higher proportion of parents of VC, compared with those of nVC, received the influenza vaccine in the last campaign, were willing to be vaccinated during the current campaign, and considered influenza a disease causing the higher number of hospitalizations. In this regard, parents of nVC considered pneumonia as the main disease causing the higher number of hospitalizations. Considering this, information materials about vaccination against influenza should mention that influenza can cause pneumonia both on its own and through complications of bacterial infection. Altogether, these could be some of the reasons influencing the decision to vaccinate their children.

The descriptive results were further confirmed by the multivariable analysis, which identified parent's influenza vaccination (or intention) during this campaign and in the previous one, as well as children's complete vaccination schedule according to their age and the administration of non-funded vaccines as factors independently associated with the probability of children being vaccinated against influenza. In this regard, it is important to mention that it has been considered that the main reason for not administering non-funded vaccines is the lack of resources (21), however, vaccine against influenza is free in Spain thus, it could be hypothesized that those who do not vaccinate their children are more reluctant to vaccination or have a lower level of health culture. The results observed in our study during the current campaign are similar to those obtained in the 2022–2023 vaccination campaign, in which a higher proportion of parents of VC had been vaccinated against influenza in the past and were willing to be vaccinated again. However, parent's influenza vaccination in the previous year was not identified as an independent factor (17). Overall, our results from the two consecutive vaccination campaigns support the idea that parents' own vaccination status and willingness influences children vaccination status. This is in line with the results from a study conducted in Canada reporting parents' characteristics associated with pediatric vaccination against influenza. The authors showed that parental current and prior influenza vaccination and the child having a history of influenza vaccination were strongly associated with children's influenza vaccination (22). Similarly, a recently published study conducted in Catalonia (Spain) surveyed parents of children under 5 years old about the intention to vaccinate their children, showing that children whose parents had ever been vaccinated or were vaccinated each year against influenza were more likely to be vaccinated (23).

Vaccinated children in our study received either the IIV or the LAIV, according to their age and the current recommendations (7–9). When comparing both groups of children, it is worth noting that more than twice of children receiving LAIV vs. IIV were vaccinated during the last campaign. However, this could be explained as children who received the LAIV were older than those who received the IIV and thus, for at least a part of the latter, the 2023–2024 campaign represented their first chance to be vaccinated against influenza. This difference was also observed during the 2022–2023 campaign (17). Furthermore, vaccination against influenza at schools may lead to a higher number of VC among those receiving the LAIV. In this regard, it is important to mention that the proportion of children reporting being vaccinated during last campaign greatly increased when compared with the previous campaign reporting on the 2021–2022 (17), in which only those at-risk children had the recommendation of vaccination.

Regarding parental motivation for vaccination in our study, the main reason was to protect the child in both groups. However, a significantly higher proportion of parents of children vaccinated with LAIV were motivated by the intention to protect other family members and the inclusion in the vaccination program. As children with LAIV were older, parents might be more concerned about the potential infection of other family members. These results are similar to those reported in other Spanish region by Burgaya-Subirana et al. (23) and those observed in the Region of Murcia during last campaign (17). It is worth noting that the proportion of parents reporting that it was recommended by their pediatrician decreased from last campaign (17). However, this could be partially explained as parents could have more information from last the 2022–2023 campaign or because parents of children of 3 and 4 years old were also informed trough the authorization letter for the vaccination at schools and a text message containing a link with the most frequently asked questions and answers about vaccination.

The main source of information about vaccination for parents of children vaccinated with IIV was the pediatrician or nurse, while parents of children vaccinated with LAIV placed the school as their main source, approximately nine times higher than those who received IIV. This difference could be explained as children with IIV are not schooled yet and they visit the pediatrician more frequently due to their younger age, and to both a higher number of consultations for minor pathologies or doubts and the scheduled healthy-child routine check-up visits. Additionally, vaccination with LAIV took place in schools and information material provided by the schools could cover all potential doubts, avoiding visits to the pediatric consultation to resolve any doubts. In this sense, considering that in our study the main source of information for children of 24–59 months old was schools and that lack of information was one of the most frequent reasons of non-vaccination in children of 6–23 months old, information about influenza vaccination could be also disseminated from kindergartens, as in the previous campaign (17), even if children are not vaccinated there.

Considering parent's satisfaction with the administered vaccines, most parents from both groups were satisfied or very satisfied, although reported satisfaction was higher for parents of children vaccinated with LAIV compared with those who received the IIV. These results are in line with our results from last campaign (17) and with previously published studies. A French study surveying parents of children vaccinated with the influenza LAIV reported that parents preferred this vaccine over the IIV (24). Similarly, an Italian study carried out in adult population (mean age of 41 years) showed that overall; the LAIV was preferred over the injection (25). Considering only those vaccinated at schools with LAIV, almost all parents were satisfied or very satisfied with vaccination. These results are in line with those reported in the pilot experience carried out in 24 public schools during the 2022–2023 seasons in which parents, teachers, and school nurses expressed a high degree of satisfaction with the vaccination program (26, 27). Overall, these results show that school vaccination against influenza is not only a feasible option but is supported by parents who are highly satisfied. Furthermore, influenza vaccination at schools has demonstrated to increase equity among children (28).

We found significant diversity in reasons for not vaccinating among parents of nVC in our study, being reticence to vaccinate the child until further experience with the vaccine, lack of recommendation from the healthcare professional, and lack of knowledge about the vaccine or the vaccination campaign the main reasons. Similar reasons were stated by parents included in the study by Burgaya-Subirana et al. (23) conducted in Spain and in ours from last campaign (17). It is worth highlighting that the proportion of parents mentioning that they do not believe in influenza vaccines in the 2023–2024 campaign has more than doubled compared with the 2022–2023 campaign (4.0% vs. 1.6%). However, this proportion is lower than that reported by Burgaya-Subirana et al. (23), in whose research 9.3% of parents distrusted any vaccine. Overall, skepticism toward vaccination should not be disregarded and more efforts should be made to raise awareness on the importance of vaccination in general and against influenza for children under 5 years in particular, which is also included in the Spanish lifelong vaccination schedule since 2023.

Vaccine hesitancy was listed by the WHO as one of the top 10 threats to global health (29). Multiple factors have been associated with vaccine hesitancy, as observed in our study and in others (30–34). In this regard, previous studies reported that parents' knowledge about influenza is related to the probability of vaccinating their children (30, 33). In addition, it is worth highlighting that one of the factors associated with influenza vaccine refusal in children is the lack of recommendation from healthcare professionals (30, 32, 33), which was also reported in our study from the 2022–2023 vaccination campaign (17) and confirmed in the present campaign. In this context, previous studies showed that healthcare professionals are the most trusted advisors for vaccination (35) and that parents who were depending on healthcare professionals as a source of information regarding influenza vaccination were less likely to report vaccine hesitancy (31). Therefore, considering our results and those previously published about healthcare professionals influence on vaccination, the visit to the pediatrician or physician represents an opportunity to promote vaccination, emphasizing the importance of vaccination.

Our study presents some limitations. First, the response rate obtained from parents invited to participate was low, especially from parents of nVC. In addition, selection bias cannot be ruled out since participation in the study was voluntary. Results should be interpreted with caution as a higher response rate could change the results obtained. However, the high number of participants included allows us to extract solid conclusions. Future studies could promote parental participation through more detailed information and incentives with the aim of improving the robustness and generalizability of the results. Second, the study was conducted in one autonomous community in Spain and therefore, the generalization of the results should be taken with caution. Third, it is important to mention that parents from VC received the survey 7 days after vaccine administration, while parents of nVC were enrolled after the end of the vaccination campaign, which might result in recall bias regarding data on vaccine effectiveness, vaccination in previous campaigns or information about the vaccine and the vaccination campaign, among others. Finally, we conducted a survey to collect information from participants. Although this method relies on participants' recall, we used a specifically designed questionnaire with a sufficient number of questions to ensure the necessary data collection.

## 5 Conclusions

The results of our study showed that parents influenza vaccination status and children's complete vaccination schedule are indicative of a higher probability of children of 6–59 months old being vaccinated against influenza. Regarding satisfaction with vaccination, parents of VC reported high satisfaction with influenza vaccination, which was even higher among parents of children vaccinated with LAIV at schools. Among non-vaccinating parents, waiting for more experience with the vaccine and lack of recommendation from healthcare professionals were the main reason for not vaccination. Overall, our results could be used to identify characteristics in parents that prevent from children vaccination against influenza. In addition, encouraging vaccination in parents through healthcare professionals or information received through the different educational institutions might help increase vaccination rates in children under the age of 5 years.

## Data Availability

The original contributions presented in the study are included in the article/Supplementary material, further inquiries can be directed to the corresponding author.
