# Supplementary material for: Vaccinating and non-vaccinating parents' attitudes toward influenza vaccination in children under 5 years old in Spain
Source: Front Public Health. 2025 Oct 1;13:1644600. doi: 10.3389/fpubh.2025.1644600 (PMC12521438; doi:10.3389/fpubh.2025.1644600)
Supplement: Supplementary file 1 [file Table_1.docx]

Supplementary Material

# Survey questions: Reasons for NOT vaccinating children aged 6-59 months in the Region of Murcia in the 2023-2024 campaign

1. In order to be able to complete the survey, please tick the first compulsory box by which you agree to participate in the study: □ I accept to participate in the study.
2. Age of your child: 6-12 months/1 year/2 years/3 years/4 years
3. Country of origin of your child: Spain/Other
4. If your answer to the previous question was ‘Other’, please indicate the child's country of birth: _____
5. Was your child born prematurely or on time?: On time (≥37 weeks)/Prematurely (<37 weeks)
6. Sex of your child: Male/Female
7. Number of sibling (not including your child): 0/1/2/>2
8. In 2023, did your child have flu or other acute infectious illness, which required medical consultation or care?: Yes (once or more often)/No
9. How often was your child prescribed antibiotics in 2023?: 0/1/2/>2
10. Why have you decided NOT to vaccinate your child against influenza in the2023-2024 campaign? (you can select more than one option): I consider flu an infection of minor importance in children <5 years/The vaccine did not demonstrate effectiveness/Lack of information about the vaccination campaign/The healthcare professional of reference did not recommend it/Influence of friends’/family’s opinion/I do not believe in vaccines/Lack of information about the vaccine/I would rather wait until further experience with the flu vaccine/I consider the flu vaccine unsafe/Inaccessibility (i.e., impossibility to get an appointment)/My son/daughter has contracted flu this year
11. Did you vaccinate your child in the last 2022-2023 flu vaccination campaign?: Yes/No
12. Has the type of vaccination your child was due for by age played a role in your decision not to vaccinate your child against influenza?: Yes, my child had to be vaccinated with the intramuscular vaccine because of their age and I didn't want to give them another injection. If it had been intranasal, yes, I would have vaccinated them/No, my child had to be vaccinated with the intramuscular vaccine because of their age, but even if it had been intranasal, I would not have vaccinated them neither/ Yes, my child had to be vaccinated with the intranasal vaccine because of their age, and I think it is less effective than the intramuscular vaccine/ No, my child's age required the intranasal vaccine and the reason I did not vaccinate them is not the route of administration
13. If you decided to vaccinate your child against the flu next season, how much influence would the fact that the vaccine was not injected have on your decision, with 1 being the least influence and 5 the most influence?: 1/2/3/4/5
14. Select the main sour of information for the flu vaccination campaign for children under 5 years: Pediatrician or pediatric nurse/Own research/Word of mouth/Press, TV or radio/Internet or social medial/School or kindergarten/Email from the school/Information sent through the school's Association of School Parents/Text message sent from the Regional Ministry of Health/Upon receiving the school vaccination letter/I have had no information about this campaign
15. Do you know anyone who has had their child vaccinated against influenza during this campaign?: Yes/No
16. If the answer above was yes, could you please rate the experience of this person, with 1 being the worst possible experience and 5 being the best?: 1/2/3/4/5
17. Are you planning to vaccinate your child against influenza next year?: Yes/No/I do not know yet
18. Is your child up to date with their immunization schedule?: Yes/No
19. Has your child received any non-funded vaccines?: Yes/No
20. Does your child have any chronic disease?: Yes/No
21. If yes, specify: Immune system deficiency/Allergic diseases/Gastrointestinal diseases/Neurological diseases/Pulmonary diseases/Kidney diseases/Cancer/Autoimmune diseases/Endocrine diseases/Genetic diseases/Heart diseases/Others

*Information about the parent or legal guardian completing the survey*

1. Age: Under 20 years/20–29 years/30–39 years/40–49 years/>50 years
2. Country of origin: Spain/Other
3. If your answer to the previous question was ‘Other’, please indicate the child's country of birth: _____
4. Sex: Male/Female
5. Education: None/Primary education/Secondary education/Higher education
6. Do you have any chronic disease?: Yes/No
7. If yes, specify: Immune system deficiency/Allergic diseases/Gastrointestinal diseases/Neurological diseases/Pulmonary diseases/Kidney diseases/Cancer/Autoimmune diseases/Endocrine diseases/Genetic diseases/Heart diseases/Others
8. Did you have a flu vaccination last season 2022-2023?: Yes/No
9. Have you had a flu vaccination this season 2023-2024?: Yes/No
10. Do you live with anyone in your household who suffers from any of the above-mentioned chronic diseases and/or is over 60 years old?: Yes/No
11. Among the childhood diseases listed below, which one do you think causes the most hospitalizations in children under 5 years of age?: Rotavirus gastroenteritis/Influenza/Meningitis/Pneumonia/Measles

# Survey questions: acceptance and satisfaction with influenza vaccination in children aged 6-59 months in the Region of Murcia (parents of children of 6-23 months old)

1. In order to be able to complete the survey, please tick the first compulsory box by which you agree to participate in the study: □ I accept to participate in the study.
2. Age of your child: 6-11 months/12-23 months
3. Country of origin of your child: Spain/Other (specify:____)
4. Was your child born prematurely or on time?: On time (≥37 weeks)/Prematurely (<37 weeks)
5. Sex of your child: Male/Female
6. Number of siblings (not including your child): 0/1/2/>2
7. In 2023, did your child have flu or other acute infectious illness, which required medical consultation or care?: Yes (once or more often)/No
8. Why have you decided to vaccinate your child for flu (you can select more than one option)?: To protect the child/To protect other members of the family/It was included in the vaccination program/It was recommended by their pediatrician or physician
9. Select the main sour of information for the flu vaccination campaign for children under 5 years: Pediatrician or pediatric nurse/Own research/Word of mouth/Press, TV or radio/Internet or social medial/School or kindergarten/Email from the school/Information sent through the school's Association of School Parents/Text message sent from the Regional Ministry of Health/Upon receiving the school vaccination letter/I have had no information about this campaign
10. Is your child up to date with their immunization schedule?: Yes/No
11. Has your child received any non-funded vaccines?: Yes/No
12. Was your child vaccinated against influenza last season?: Yes/No
13. Does your child have any chronic disease?: Yes/No
14. If yes, specify: Immune system deficiency/Allergic diseases/Gastrointestinal diseases/Neurological diseases/Pulmonary diseases/Kidney diseases/Cancer/Autoimmune diseases/Endocrine diseases/Genetic diseases/Heart diseases/Others

*Information about the parent or legal guardian completing the survey*

1. Age: Under 20 years/20–29 years/30–39 years/40–49 years/>50 years
2. Country of origin: Spain/Other (specify:____)
3. Sex: Male/Female
4. Education: None/Primary education/Secondary education/Higher education
5. Do you have any chronic disease?: Yes/No
6. If yes, specify: Immune system deficiency/Allergic diseases/Gastrointestinal diseases/Neurological diseases/Pulmonary diseases/Kidney diseases/Cancer/Autoimmune diseases/Endocrine diseases/Genetic diseases/Heart diseases/Others
7. Did you have a flu vaccination last season?: Yes/No
8. Are you going to get or have you had a flu vaccination this season?: Yes/No
9. Do you live with anyone in your household who suffers from any of the above-mentioned chronic diseases and/or is over 60 years old?: Yes/No
10. Among the childhood diseases listed below, which one do you think causes the most hospitalizations in children under 5 years of age?: Rotavirus gastroenteritis/Influenza/Meningitis/Pneumonia/Measles

*Questionnaire for parents/legal guardians on acceptability/satisfaction after administration of intramuscular inactivated vaccine (Influvac^®^ Tetra) in children aged 6 to 23 months*

1. Has your child had any additional vaccinations on the same day as the flu vaccination?: Yes/No
2. Has your child experienced any adverse effects related to the de flu vaccine within 7 days of its administration?: Yes/No
3. If yes, have any of these adverse effects interfered with their daily life?: Yes/No
4. If yes, have any of these adverse effects required treatment?: Yes/No
5. Are you satisfied with your child's flu vaccination?: 1/2/3/4/5
6. Do you intend to vaccinate your child again next flu season with this vaccine?: Yes/No/Maybe
7. Following your experience with the vaccination of your child, would you recommend to your family members’ and friends’ children to be vaccinated against influenza?: Yes/No
8. If no, indicate the main reason: Due to secondary effects/It is not considered to be a very effective vaccine/Vaccination against influenza is not important/Other
9. If the vaccine next season was intranasal instead of injected, would you prefer this other option?: Definitely/Probably/Indifferent/Probably not/No/ as advised by the pediatrician or nurse

# Survey questions: acceptance and satisfaction with influenza vaccination in children aged 6-59 months in the Region of Murcia (parents of children of 24-59 months old)

1. In order to be able to complete the survey, please tick the first compulsory box by which you agree to participate in the study: □ I accept to participate in the study.
2. Age of your child: /2 years/3 years/4 years
3. Country of origin of your child: Spain/Other (specify:____)
4. Was your child born prematurely or on time?: On time (≥37 weeks)/Prematurely (<37 weeks)
5. Sex of your child: Male/Female/Non-binary
6. Number of siblings (not including your child): 0/1/2/>2
7. In 2023, did your child have flu or other acute infectious illness, which required medical consultation or care?: Yes (once or more often)/No
8. Why have you decided to vaccinate your child for flu (you can select more than one option)?: To protect the child/To protect other members of the family/It was included in the vaccination program/It was recommended by their pediatrician or physician
9. Select the main sour of information for the flu vaccination campaign for children under 5 years: Pediatrician or pediatric nurse/Own research/Word of mouth/Press, TV or radio/Internet or social medial/School or kindergarten/Email from the school/Information sent through the school's Association of School Parents/Text message sent from the Regional Ministry of Health/Upon receiving the school vaccination letter/I have had no information about this campaign
10. Is your child up to date with their immunization schedule?: Yes/No
11. Has your child received any non-funded vaccines?: Yes/No
12. Was your child vaccinated against influenza last season?: Yes/No
13. Does your child have any chronic disease?: Yes/No
14. If yes, specify: Immune system deficiency/Allergic diseases/Gastrointestinal diseases/Neurological diseases/Pulmonary diseases/Kidney diseases/Cancer/Autoimmune diseases/Endocrine diseases/Genetic diseases/Heart diseases/Others

*Information about the parent or legal guardian completing the survey*

1. Age: Under 20 years/20–29 years/30–39 years/40–49 years/>50 years
2. Country of origin: Spain/Other (specify:____)
3. Sex: Male/Female/Non-binary
4. Education: None/Primary education/Secondary education/Higher education
5. Do you have any chronic disease?: Yes/No
6. If yes, specify: Immune system deficiency/Allergic diseases/Gastrointestinal diseases/Neurological diseases/Pulmonary diseases/Kidney diseases/Cancer/Autoimmune diseases/Endocrine diseases/Genetic diseases/Heart diseases/Others
7. Did you have a flu vaccination last season?: Yes/No
8. Are you going to get or have you had a flu vaccination this season?: Yes/No
9. Do you live with anyone in your household who suffers from any of the above-mentioned chronic diseases and/or is over 60 years old?: Yes/No
10. Among the childhood diseases listed below, which one do you think causes the most hospitalizations in children under 5 years of age?: Rotavirus gastroenteritis/Influenza/Meningitis/Pneumonia/Measles

*Questionnaire for parents/legal guardians on acceptability/satisfaction after administration of the intranasal inactivated vaccine (Fluenz^®^ Tetra) in children aged 24 to 59 months*

1. Has your child had any additional vaccinations on the same day as the flu vaccination?: Yes/No
2. Has your child experienced any adverse effects related to the de flu vaccine within 7 days of its administration?: Yes/No
3. If yes, have any of these adverse effects interfered with their daily life?: Yes/No
4. If yes, have any of these adverse effects required treatment?: Yes/No
5. Are you satisfied with your child's flu vaccination?: 1/2/3/4/5
6. Do you intend to vaccinate your child again next flu season with this vaccine?: Yes/No/Maybe
7. Following your experience with the vaccination of your child, would you recommend to your family members’ and friends’ children to be vaccinated against influenza with the intranasal vaccine?: Yes/No
8. If no, indicate the main reason: Due to secondary effects/It is not considered to be a very effective vaccine/Vaccination against influenza is not important/Other
9. Where has your child been vaccinated against influenza in the 2023-2024 season?: At the usual health center or vaccination station/At school
10. Have you had the opportunity to vaccinate your child at school?: Yes/No
11. If your child was vaccinated at school, please express your level of satisfaction, with 1 being the lowest possible and 5 being the highest possible score: 1/2/3/4/5
12. If your child was vaccinated at school, which of the following issues do you consider to be the most important in school vaccination (you can only choose one option)?: It makes the process easier because I don't have to make an appointment/They get vaccinated with their peers/ They behave better than they would at the usual health center or vaccination station
13. If your child was born in 2019 or 2020, they could have been vaccinated at school, if they did not, can you indicate your reasons (you can select more than one option)?: I am afraid of not being present/ I have no problem taking them to the usual health center or vaccination station/ Regardless of my presence, I think it is better to vaccinate them at the usual health center or vaccination station/ My child cannot be vaccinated at school because they have one of the contraindications specified in the letter/I have not received information about flu vaccination at school/ My child is not attending school/Other (specify:____)
14. If the vaccine had been injected instead of intranasal, would you still have vaccinated your child anyway?: Yes/No
15. If your child has been vaccinated at school with the intranasal vaccine, would you have vaccinated them at school if the vaccination had been done with the intramuscular vaccine instead of the intranasal vaccine?: Yes/No
